# Overexpression of the Glutathione Peroxidase 5 (*RcGPX5*) Gene From *Rhodiola crenulata* Increases Drought Tolerance in *Salvia miltiorrhiza*

**DOI:** 10.3389/fpls.2018.01950

**Published:** 2019-01-09

**Authors:** Lipeng Zhang, Mei Wu, Yanjiao Teng, Shuhang Jia, Deshui Yu, Tao Wei, Chengbin Chen, Wenqin Song

**Affiliations:** Department of Genetics, College of Life Sciences, Nankai University, Tianjin, China

**Keywords:** *Rhodiola crenulata*, glutathione peroxidase, *Salvia miltiorrhiza*, drought, secondary metabolites

## Abstract

Excessive cellular accumulation of reactive oxygen species (ROS) due to environmental stresses can critically disrupt plant development and negatively affect productivity. Plant glutathione peroxidases (GPXs) play an important role in ROS scavenging by catalyzing the reduction of H_2_O_2_ and other organic hydroperoxides to protect plant cells from oxidative stress damage. *RcGPX5*, a member of the GPX gene family, was isolated from a traditional medicinal plant *Rhodiola crenulata* and constitutively expressed in *Salvia miltiorrhiza* under control of the CaMV 35S promoter. Transgenic plants showed increased tolerance to oxidative stress caused by application of H_2_O_2_ and drought, and had reduced production of malondialdehyde (MDA) compared with the wild type. Under drought stress, seedlings of the transgenic lines wilted later than the wild type and recovered growth 1 day after re-watering. In addition, the reduced glutathione (GSH) and total glutathione (T-GSH) contents were higher in the transgenic lines, with increased enzyme activities including glutathione reductase (GR), ascorbate peroxidase (APX), and GPX. These changes prevent H_2_O_2_ and O_2_^-^ accumulation in cells of the transgenic lines compared with wild type. Overexpression of *RcGPX5* alters the relative expression levels of multiple endogenous genes in *S. miltiorrhiza*, including transcription factor genes and genes in the ROS and ABA pathways. In particular, *RcGPX5* expression increases the mass of *S. miltiorrhiza* roots while reducing the concentration of the active ingredients. These results show that heterologous expression of *RcGPX5* in *S. miltiorrhiza* can affect the regulation of multiple biochemical pathways to confer tolerance to drought stress, and *RcGPX5* might act as a competitor with secondary metabolites in the *S. miltiorrhiza* response to environmental stimuli.

## Introduction

Plant growth and seed production are subjected to various environmental stresses such as drought, salinity, and temperature extremes (Kilian et al., 2007). Drought often induces osmotic stress which can perturb the homeostasis and ion distribution in plant cells (Wang et al., 2003). Plants have evolved a series of physiological and biochemical processes that allow them to respond and adapt to environmental stresses such as drought (Shinozaki et al., 2003). When the moisture content decreases, the water potential in the cell is lower than that of the extracellular environment, which results in water efflux from the cell. Many plants accumulate organic osmolytes and zwitterionic alkylamines to maintain osmotic potential and cell expansion (Hare et al., 1998; Selvaraj et al., 2017). In addition, plants also employ molecular control mechanisms for drought tolerance. Some transcription factors such as CBF/DREB (Ravikumar et al., 2014), MYB (Abe, 2002), and NAC (Hu et al., 2006) can be induced by drought stress and modify global gene expression to cope with drought (Zhang et al., 2017). These processes are complex and involve many biochemical pathways that include calcium signaling and biosynthesis of the plant hormone abscisic acid (Knight et al., 1997; Kang et al., 2002; Yunta et al., 2011).

Drought also causes a type of oxidative stress by generating reactive oxygen species (ROS), such as superoxide radical, hydrogen peroxide, and hydroxyl radical (Munne-Bosch et al., 2013). However, ROS are inherent compounds that are widespread in plants. Under normal conditions, biological processes such as photosynthetic electron transport in the chloroplast and respiration in mitochondria produce ROS (Suzuki et al., 2012). Thus, ROS are not only toxic for aerobic organisms but also function as important signal transduction molecules to mediate plant cell responses to abiotic stresses (Miller et al., 2008). However, excess ROS are harmful to cells and can damage the plasma membrane and oxidize molecules such as nucleic acids and proteins (Csiszár et al., 2012). Plants expend energy to scavenge ROS, which is necessary for plant stress tolerance. In order to maintain low cellular ROS concentrations, scavenging occurs through the activity of enzymes such as catalase (CAT), superoxide dismutase (SOD), peroxiredoxins (POD), glutathione peroxidase (GPX), ascorbate peroxidase (APX), and also via several non-enzymatic antioxidant compounds such as ascorbate and glutathione (Kang et al., 2004; Chang et al., 2009).

Glutathione peroxidase is a key component of the glutathione-ascorbate cycle to reduce the accumulation of H_2_O_2_ by oxidizing reduced glutathione (GSH) to the disulfide form (GSSG) (Mittova et al., 2003). Plant GPX protein sequences show high sequence similarities with mammalian phospholipid hydroperoxide GPX4 (Faltin et al., 2010). However, plant GPXs contain three conserved non-selenium Cys residues in the active sites and catalyze H_2_O_2_ using thioredoxin as an electron donor instead of GSH, which is a different mechanism than that used by animal GPXs (Koh et al., 2007; Islam et al., 2015). The major functions of plant GPXs are to prevent programed cell death from oxidative damage due to stress (Chen et al., 2004). The plant GPX protein family consists of multiple isoenzymes located in different subcellular compartments that have distinct expression patterns with respect to tissues and developmental stages (Gao et al., 2014). In addition, GPXs can be induced by abiotic stresses such as drought, salt, and cold (Yoshimura et al., 2004; Miao et al., 2006; Kim et al., 2014). Also, transgenic plants overexpressing *GPX* genes show tolerance to these stresses (Gaber et al., 2012; Diao et al., 2014), which makes them excellent candidate genes for genetic engineering of drought tolerant crops.

*Salvia miltiorrhiza* (Chinese sage or red sage) is an herbaceous perennial in the botanical family Lamiaceae that has been used in traditional Chinese medicine for thousands of years (Yang N. et al., 2017). According to the pharmacopeia, the roots and rhizomes contain several secondary metabolites, salvianolic acids and tanshinones that are useful for the treatment of dysmenorrhoea, amenorrhoea, and cardiovascular diseases (Pharmacopoeia, 2010; Ma et al., 2012). Therefore, the discovery of many more economic and medicinal uses for *S. miltiorrhiza* has increased the demand in recent years for this herb. However, because of serious environmental damage, especially from drought due to global climate change, the planting areas and yield of *S. miltiorrhiza* have been significantly reduced (Wei et al., 2016a). In order to meet the increasing demand, genetic engineering strategies have been developed to engineer tolerance to environmental stresses or increase the contents of the active ingredients (Ding et al., 2017; Zhou et al., 2018). However, most of the published studies have focused on increasing the contents of phenolic acids and tanshinones, while there is little research describing stress tolerance in *S. miltiorrhiza* (Wei et al., 2017).

*Rhodiola crenulata* (Crassulaceae) is also a popular medicinal plant that is widely used for its antioxidant, anti-tumor, antidepressive, cardioprotective, hepatoprotective, and immunostimulant properties (Qu et al., 2012). *R. crenulata* occurs naturally in highland regions at altitudes of 3500–5000 m in the Qing-Tibet plateau, northern Yunnan, and western Sichuan provinces (Fu et al., 2017). The alpine environment is very different from lower altitude areas, where plants are exposed to cold temperatures, low humidity, reduced oxygen levels, longer day lengths, and strong UV irradiation (Zhang et al., 2014a,b). Thus, we can speculate that *R. crenulata* is able to remove high levels of ROS that are caused by exposure to oxidative stresses in the extreme alpine environment. Although there have been reports that overexpression of GPX can confer tolerance to multiple stresses, whether *RcGPX5* is able to protect plants from drought-related injury has not been analyzed. In this study, we isolated a cDNA fragment from *R. crenulata* containing the entire open reading frame of a GPX and overexpressed it in transgenic plants of *S. miltiorrhiza*. We then demonstrated the improved survival of the transgenic plants after exposure to H_2_O_2_ and drought stress conditions. Furthermore, after 5 months growing under natural conditions, we measured the contents of glutathione and active ingredients in the roots. The results showed that overexpressing *RcGPX5* improved tolerance to drought in *S. miltiorrhiza* and increased the yield of roots with negative effects on the accumulation of secondary metabolites.

## Materials and Methods

### Plant Transformation Vector Construction

A gene that encodes a predicted protein with high sequence similarity to *Arabidopsis* GPX5 was obtained from the transcriptome unigene assembly database of *R. crenulata* (unpublished). This unigene was named *RcGPX5* and has been confirmed to carry the entire open reading frame of a GPX. The *RcGPX5* cDNA was isolated from RNA extracted from *R. crenulata* leaves. Total RNA extraction and first-strand cDNA synthesis were performed using the Easy RNA extraction kit and the First chain cDNA synthesis kit (Promega, Beijing China), respectively. PCR amplification of *RcGPX5* was conducted using a high-fidelity thermostable DNA polymerase (Takara, Japan) and primers containing the restriction sites *Nco*I and *Bst* EII. The amplification product was inserted into the *pEASY*-T1 vector (TransGen Biotech, Beijing, China) and the DNA insert was sequenced using Sanger technology (Biotech, Shanghai, China). To construct the recombinant plant expression vector, the *RcGPX5* cDNA fragment was cloned into the binary vector pCAMBIA3301 under control of the CaMV35S constitutive promoter by double digestion with *Nco*I and *Bst* EII. The resulting plasmid vectors were transformed into *Escherichia coli* DH5α and *Agrobacterium tumefaciens* EHA105 using a standard heat-shock method.

### Generation and Molecular Characterization of Transgenic Plants

To generate transgenic plants of *S. miltiorrhiza*, we used the *Agrobacterium*-mediated leaf-disk method with some modifications (Wang et al., 2013). Actively growing leaves were cut into 1-cm squares and immersed in 100 mL of Agrobacterium cell suspension (OD_600_ = 0.6–0.8) in liquid MS medium for 25 min. The leaf squares were then transferred to MS medium containing 20 μmol/L acetosyringone for co-culture in the dark. After 2 days, the explants were collected and rinsed with sterile water six times. The explants were blotted dry and placed on MS medium containing 1.0 mg/L 6-benzyl-aminopurine (BAP), 1.0 mg/L naphthalene acetic acid (NAA), 0.8 mg/L basta, and 200 mg/L cefotaxime (cef) for selection. When multiple shoots had grown from the explants, the shoots were removed and transferred onto fresh MS medium containing 0.2 mg/L indole-3-butytric acid (IBA), 0.8 mg/L basta, and 200 mg/L cef.

Total RNA was extracted from wild-type (WT) and regenerated transgenic seedlings using the Easy RNA extraction kit (Promega, Beijing China). The molecular identification of the transgenic lines was performed by semi-quantitative RT-PCR with specific primers. The primer pairs 35S-F/RcGPX5-R (35S-F: AACAGAACTCGCCGTAAAG, RcGPX5-R: GTTGCACGGGAAAGCCAATAT) and RcGPX5-F/R (RcGPX5-F/R: ATGGGTGCTTCCCCTTCTGTC/TTACTCATCTTTTCCCAGTGC) were used to select valid transgenic lines. The primer pair RcGPX5-qF/R (RcGPX5-qF/R: GAGAAATCCATCCACGATTTCAC/GTTGCACGGGAAAGCCAATAT) was used in real-time RT-PCR assays to select the high expression lines. The *S. miltiorrhiza actin* gene (SmActin-F/R: GGTGCCCTGAGGTCCTGTT/AGGAACCACCGATCCAGACA) was used as the reference to normalize gene expression. For the next experiments, T0 generation transgenic lines were used in this paper.

### Stress Treatments

Seedlings of the wild type and transgenic lines of equal sizes with the roots removed were transferred to fresh solid MS medium in a growth chamber for 25–30 days. The plantlets were then moved to flower pots containing the same quantity of sterile solid medium and cultured for 20 days at 25°C with a 16 h/8 h photoperiod. Prior to the H_2_O_2_ and drought treatments, the plants were watered. For H_2_O_2_ stress, detached leaves at the same developmental stages were cut into squares and soaked in 0.5X liquid MS media containing 50 μmol/L H_2_O_2_ and 0.1% Tween 20 on a shaker at 100 rpm in the dark for 8 h. Whole leaves were also treated under the same conditions but at an H_2_O_2_ concentration of 10 mmol/L (Yoshimura et al., 2004; Passaia et al., 2013). For drought treatment, the well-watered seedlings were treated as described in a previous report (Wei et al., 2016b). Plants were exposed to drought for 14 days, after which all plants were re-watered.

### Determining the Physiological Indices of Leaves and the Active Ingredients in Roots

For all the physiological indices examined in leaves and roots, we performed a minimum of three biological replicates and three measurements by 752-UV spectrophotometry. The contents of chlorophyll a, chlorophyll b, and xanthophylls were determined according to the method of Richardson et al. (2002). The malondialdehyde (MDA) content was assayed by the thiobarbituric acid (TBA) method (Wu et al., 2014). The O_2_^-^ and H_2_O_2_ contents as well as the superoxide dismutase (SOD) and L-ascorbate peroxidase (APX) activities were assayed using previously described methods (Rosa et al., 2010; Yang M. et al., 2017). The glutathione (GSH), total glutathione (T-GSH), and ascorbic acid (ASA) contents, and the GPX and glutathione reductase (GR) activities were measured using assay kits, following the manufacturer’s instructions (Nanjing Jiancheng Bioengineering Institute, Nanjing, China).

The levels of the active ingredients in the roots, salvianolic acids and tanshinones, were determined using a Shimadzu LC-20AT HPLC system as previously described (Ge et al., 2015; Hao et al., 2015). Tanshinone metabolites include tanshinone IIA, tanshinone I, and cryptotanshinone. The assayed roots came from plants of the WT and transgenic lines after 5 months of growth in the experimental field under natural environmental conditions. The materials were treated and quantified with authentic standards (Pharmacopoeia, 2010). All data are presented as the means ± standard error (SE) of at least three replicates. Student’s *t*-test was used to test the significance of differences compared to pre-treatment materials. Asterisks (^∗^ and ^∗∗^) indicate significant differences between the controls and transgenic plants at *p* < 0.05 and 0.01, respectively.

### Quantitative Real Time PCR (qRT-PCR)

Gene expression levels were measured by qRT-PCR for genes in several metabolic pathways including the ROS pathway and the tanshinone and salvianolic acid biosynthesis pathways, as well as for other genes related to drought stress. The PCR primers were designed using Primer Premier 5 software (Premier Biosoft) based on the transcriptome assembly unigene database from our laboratory (Wei et al., 2018) and sequences downloaded from NCBI (Supplementary Tables S1, S2). The qRT-PCR reactions were conducted as previously described (Yang M. et al., 2017) using SYBR Green I (Roche, China), and the internal reference gene was *SmACTIN*. The gene expression levels were calculated by the 2^-ΔΔCt^ method with three biological replicates (Livak and Schmittgen, 2001).

## Results

### Generation and Molecular Characterization of *RcGPX5* Transgenic Lines of *S. miltiorrhiza*

**FIGURE 1:**
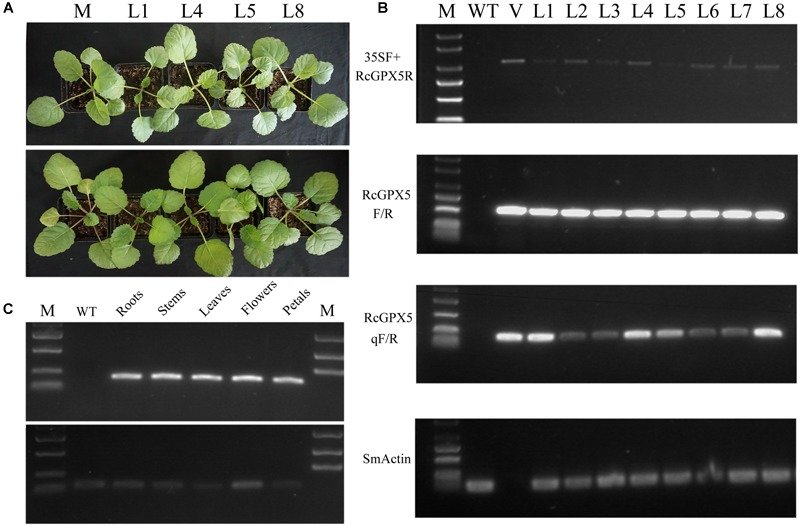
Identification and characterization of transgenic *Salvia miltiorrhiza* plants expressing *RcGPX5*. **(A)** The identities of wild type and transgenic lines are abbreviated as follows: WT, wild type; L1/L4/L5/L8, four individual plants from positive transgenic lines. **(B)** RT-PCR analysis of primary transformants using several primer combinations. Using *SmACTIN* as control, PCR amplification assays were conducted using cDNA from WT and putative transgenic lines produced by transformation with the plasmid vector p35SS::RcGPX5. L1–L8 indicate the putative transgenic lines. V is the plasmid vector; M is the DNA marker. 35SF is the forward primer that anneals to the 35S promoter and RcGPX5R is the reverse primer that anneals to a site in *RcGPX5*. The primer pair RcGPX5F/R was used to amplify the entire coding region of *RcGPX5*. The primer pair RcGPXqF/R was used for semi-quantitative real-time PCR to analyze the relative expression levels of *RcGPX5* in the different transgenic lines. **(C)** RT-PCR analysis to quantify the tissue-specific expression of *RcGPX* in the L1 transgenic plantlets. The bottom was amplification production of Smactin primer and the top was qRcGPX5.

Using Agrobacterium-mediated transformation, many primary shoots were regenerated from the leaf squares and shoots in culture. The shoots grew well after transfer to solid medium supplemented with Basta herbicide and hormones for selection of valid transgenic seedlings. The phenotypes of all regenerated seedlings were the same as in the WT (Figure 1A). For the well-rooted plantlets of WT and the transgenic lines, molecular identification was conducted by PCR for eight selected individuals and WT with different primer combinations using the vector as the positive control (Figure 1B). *SmACTIN* was used as the internal reference gene, and all amplified fragments were of the expected sizes predicted from the cDNA. For the recombination primer pairs 35S-F/RcGPX5-R and RcGPX5-F/R, the positive bands amplified were 1042 and 529 bp, respectively, using the vector and cDNA from the transgenic lines as templates. Furthermore, the results of semi-quantitative PCR using primer pair RcGPX5-qF/R showed that transgenic lines 1, 4, 5, and 8 had higher *RcGPX5* expression levels (Supplementary Figure S1A). In addition, the *RcGPX5* gene was highly expressed under control of a constitutive promoter, and the expression levels were similar in the roots, stems, leaves, flowers, and petals of line 1 (Figure 1C and Supplementary Figure S1B).

### Effects of Oxidative Damage Caused by H_2_O_2_ Stress

**FIGURE 2:**
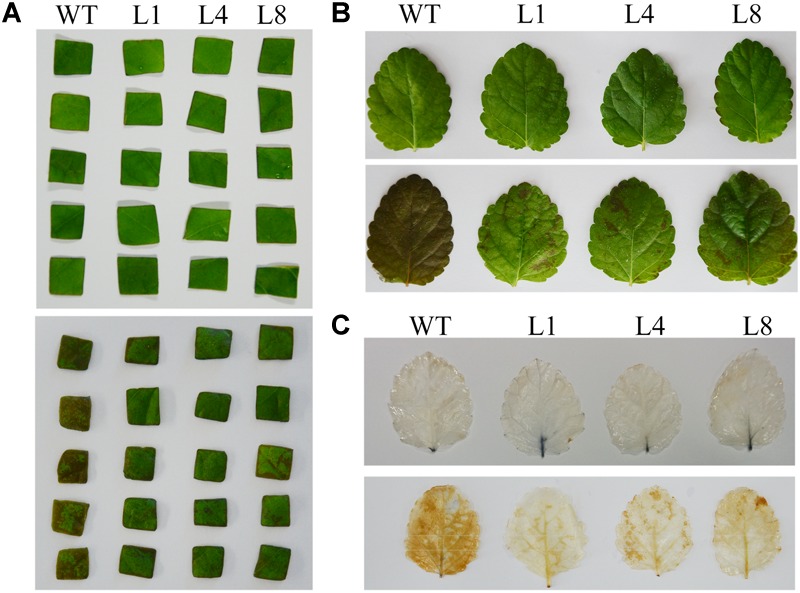
Effect of exogenous H_2_O_2_ on leaf disks and whole leaves of WT and three transgenic lines of *S. miltiorrhiza*. **(A)** Leaf squares after H_2_O_2_ treatment. The leaf pieces from WT and the transgenic lines (L1, L4, and L8) were divided into similar size groups. The squares were immersed in 0.5X liquid MS media containing 50 μmol/L H_2_O_2_ and 0.1% Tween 20. The top panel shows the control condition and the below shows the treatment condition. **(B)** Whole leaves after H_2_O_2_ treatment. Leaves were treated with 10 mmol/L H_2_O_2_. **(C)** Whole leaves were from the WT and transgenic lines were stained with diaminobenzidine (DAB). The leaves were treated with H_2_O_2_ prior to DAB staining. The brown staining shows sites of H_2_O_2_ accumulation and indicates the relative degree of leaf damage.

Because a major function of GPX is to reduce H_2_O_2_ levels, detached whole leaves and cut leaf squares from the WT and transgenic lines were immersed in treatment buffer to assess their tolerance to exogenous H_2_O_2_. Both squares and whole leaves showed damage in response to H_2_O_2_ exposure, but the transgenic lines differed from WT in that they had smaller necrotic lesions and an overall healthier, greener appearance (Figures 2A,B). The damaged regions and residual H_2_O_2_ in the leaves could be visualized by histochemical staining with 3,3-diaminobenzidine (DAB). As shown in Figure 2C, the WT leaves showed darker and more extensive brown staining than did the transgenic lines, which indicated that the *RcGPX5*-expressing transgenic lines accumulated less H_2_O_2_ and were able to endure H_2_O_2_ stress much better than the WT.

**FIGURE 3:**
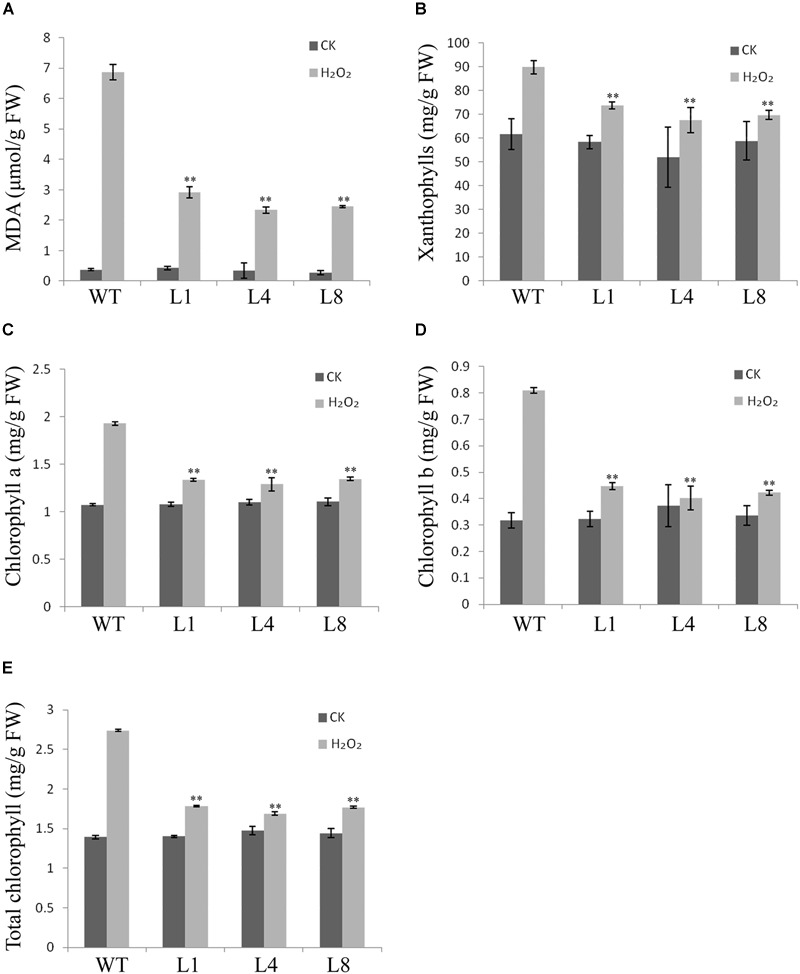
Effects of H_2_O_2_ stress on malondialdehyde (MDA) and pigment contents in WT and three transgenic lines of *S. miltiorrhiza*. Contents of **(A)** MDA, **(B)** xanthophylls, **(C)** chlorophyll a, **(D)** chlorophyll b, **(E)** total chlorophyll. The values in **(E)** represent the sum of chlorophyll a and b. Bars represent the mean ± SE of three independent experiments. ^∗∗^ indicate significant differences at *p* < 0.01 compared with WT.

Excessive H_2_O_2_ can induce lipid peroxidation, which damages cell membranes and is a marker for membrane perturbation and inactivation of membrane proteins, leading ultimately to cell death. Malondialdehyde (MDA) content is often used as an index of lipid peroxidation. As expected, following H_2_O_2_ treatment, MDA concentration was distinctly higher in WT compared with the transgenic lines (Figure 3A). However, the contents of chlorophyll a, chlorophyll b, and xanthophylls in the WT were also higher than in the transgenic lines (Figures 3B–E). These values might demonstrate that *RcGPX5* overexpressed in leaves can scavenge more H_2_O_2_ and protect cell membranes to prevent intracellular water loss from the oxidative damage caused by exogenous H_2_O_2_.

### Effects of Oxidative Damage Caused by Drought Stress

**FIGURE 4:**
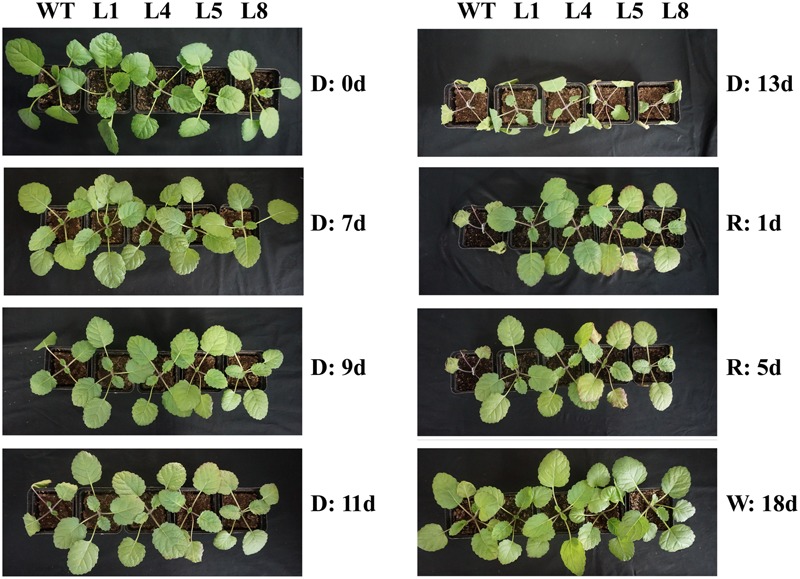
Morphological changes in plants of WT and four transgenic lines of *S. miltiorrhiza* in response to drought stress. D, Drought; R, Re-watered; W, well-watered; WT, wild type; L1/L4/L5/L8, transgenic lines that express *RcGPX5*.

To investigate whether high-level expression of *RcGPX5* from the constitutive CaMV 35S promoter in *S. miltiorrhiza* could promote tolerance to drought, plantlets of WT and four of the transgenic lines (L1, L4, L5, and L8) of similar sizes were exposed to drought stress for 13 days (Figure 4). During the drought treatment, all plantlets appeared normal on day 7 but began to show differences on day 9. The leaves of WT showed wilting and continued losing water during the subsequent 4 days. However, the transgenic line plants showed better growth status at 9 days of treatment and did not begin to wilt until day 11. After 13 days of drought, all plantlets were re-watered, and only the transgenic line plants had recovered by the next day.

**FIGURE 5:**
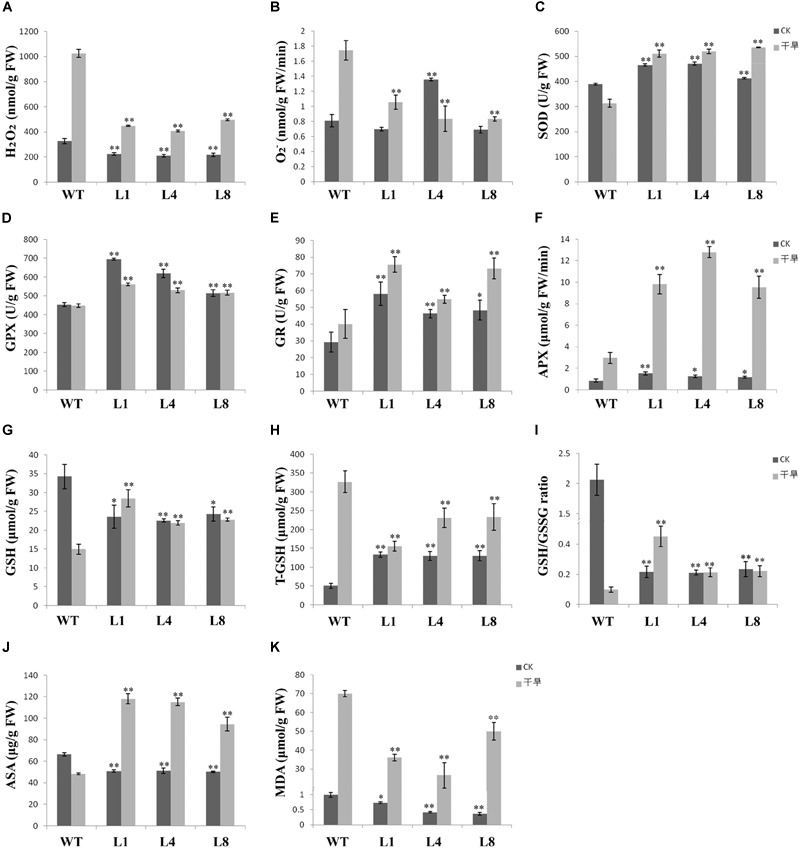
Effects of drought stress on metabolite contents and enzyme activities in WT and three transgenic lines of *S. miltiorrhiza*. Leaves from WT and transgenic plants were used for the physiological measurements after 11 days of drought stress. **(A)** H_2_O_2_ accumulation; **(B)** O_2_^-^ production rate; **(C)** superoxide dismutase (SOD) enzyme activity; **(D)** glutathione peroxidase (GPX) enzyme activity; **(E)** glutathione reductase (GR) enzyme activity; **(F)** L-ascorbate peroxidase (APX) enzyme activity; **(G)** reduce glutathione (GSH) content; **(H)** total glutathione (T-GSH) content; **(I)** GSH/GSSG ratio. The oxidized glutathione (GSSG) content was calculated using formula [GSSG = 1/2(Total GSH-GSH)]; **(J)** ascorbate (ASA) content; **(K)** malondialdehyde (MDA) content. The physiological measurements were made from leaves of WT and the transgenic lines under well-watered conditions. Bars represented the mean ± SE of three independent experiments. ^∗^ and ^∗∗^ represent significant differences at *p* < 0.05 and *p* < 0.01 compared to WT.

To determine whether the transgenic plants could reduce ROS accumulation and cellular damage caused by drought, we measured the concentrations of H_2_O_2_ and O_2_^-^ (Figures 5A,B). Following drought treatment, the O_2_^-^ and H_2_O_2_ concentrations were both higher in WT than in the transgenic plants. The MDA contents were also significantly higher in WT, which demonstrated that the level of damage to the cell membranes in WT was much higher than in the transgenic lines (Figure 5K). However, the activities of several ROS antioxidant enzymes were lower in WT compared with transgenic lines under drought conditions. SOD activity and the activities of key enzymes of the glutathione-ascorbate cycle, including GR, GPX, and APX, were all higher in the transgenic lines compared to WT, and the activities of SOD, GR, and APX were all elevated in response to drought (Figures 5C–F). These enzymes are crucial for ROS scavenging and served to protect the cells and the organism from oxidative damage.

Glutathione peroxidase catalyzes the oxidation of reduced monomeric GSH to GSSG (glutathione disulfide). To determine whether a high levels of GPX enzyme activity could have an influence on the glutathione-ascorbate cycle, we measured the antioxidant compounds in the WT and transgenic plantlets. The results showed that, under normal conditions, total GSH levels in the transgenic lines were higher than in WT; however, the contents of GSH and ASA, and the GSH/GSSG ratio were less than in WT (Figures 5G–J). Compared with the well-watered condition, drought stress increased the total GSH levels in all lines, and the greatest increase was in WT (Figure 5H). GSH content was decreased in the WT and showed only slight changes in the transgenic lines (Figure 5G). The values for the GSH/GSSG ratios were increased in the transgenic lines under drought compared to the WT, which is the inverse of the situation in the well-watered WT plants (Figure 5I). These results showed that under control conditions, the *RcGPX5*-expressing transgenic lines had an increased total glutathione pool and also elevated activities of enzymes in the glutathione-ascorbate cycle, which contributed to the survival of the plants under drought stress.

### *RcGPX5* Overexpression Alters the Expression Levels of Different Genes in *S. miltiorrhiza*

**FIGURE 6:**
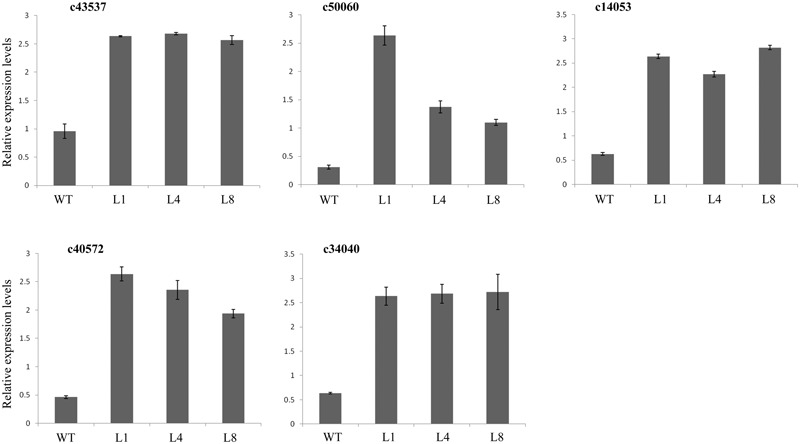
Relative expression levels of genes in the ROS pathways. qRT-PCR primers were designed from gene sequences in the *S. miltiorrhiza* transcriptome. Genes were annotated as encoding catalase (CAT), c43537; glutathione synthetase (GSH II), c50060; L-ascorbate peroxidase (APX), c14053; monodehydroascorbate reductase (MDHAR), c40572; and glutathione reductase (GR), c34040. mRNA levels were normalized with respect to *SmACTIN*. Data represent the means ± SE of at least three replicates.

**FIGURE 7:**
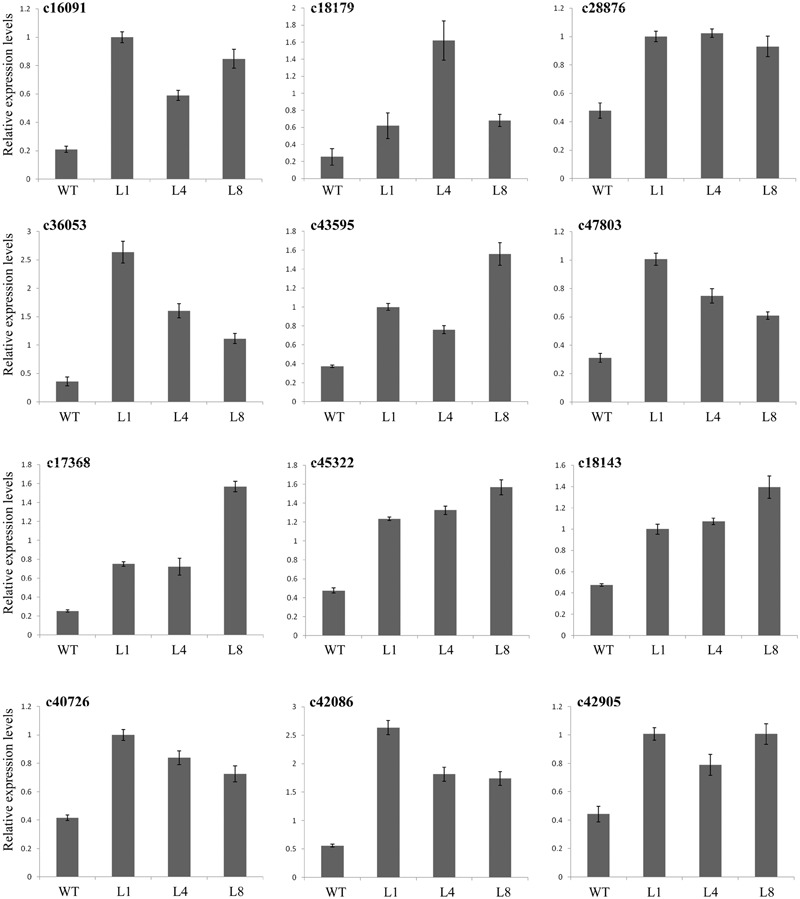
Expression levels of genes related to drought stress. The materials were used with leaves of WT and transgenic lines under control and drought stress. Primers were designed by the sequences from transcriptome. c16091, c18179, c28876, c36053, c43595, and c47803 were annotated as encoding proteins with functions in energy production and conversion or photosystem pathways; c17368 was annotated as a gene for transcription factor dehydration-responsive element-binding protein 1s (DREB1s)/C-repeat-binding factor (CBF); c45322 was annotated as a protein phosphatase 2C ABI2 gene; c18143, c40726, c42086, and c42905 were annotated as genes with functions related to the stress response. mRNA levels were normalized with respect to *SmACTIN*. Data represent the means ± SE of at least three replicates.

To understand the molecular mechanism underlying drought stress, we chose candidate genes associated with several different metabolic pathways from the transcriptome assembly unigene database of *S. miltiorrhiza* (Wei et al., 2018). We measured the expression levels of the candidate genes in the WT and transgenic lines expressing *RcGPX5* using qRT-PCR. All the genes showed differential expression in the transgenic lines compared to WT (Figures 6, 7). The key enzyme genes of the ROS pathway, such as catalase (Unigene ID: c43537), APX (Unigene ID: c14953), GR (Unigene ID: c34040), glutathione synthesis (Unigene ID: c50060), and monodehydroascorbate reductase (Unigene ID: c40572) were all upregulated in the transgenic lines (Figure 6). *RcGPX5* expression also affected the ABA signaling pathway by increasing the relative expression of protein phosphatase 2C (ABI2, Unigene ID: c45322). The well-known stress-inducible gene CBF (Unigene ID: 17368) also showed increased expression due to *RcGPX5* overexpression and should confer drought tolerance. In addition, several unigenes related to energy production and conversion, photosystems or stress responsive pathways were also upregulated (Figure 7). The high expression levels of these genes in WT leaves under control conditions could provide the potential tolerance for transgenic plants prior to drought stress.

### Overexpression of *RcGPX5* Improves Biomass and Glutathione Contents in Transgenic Plants of *S. miltiorrhiza*

**FIGURE 8:**
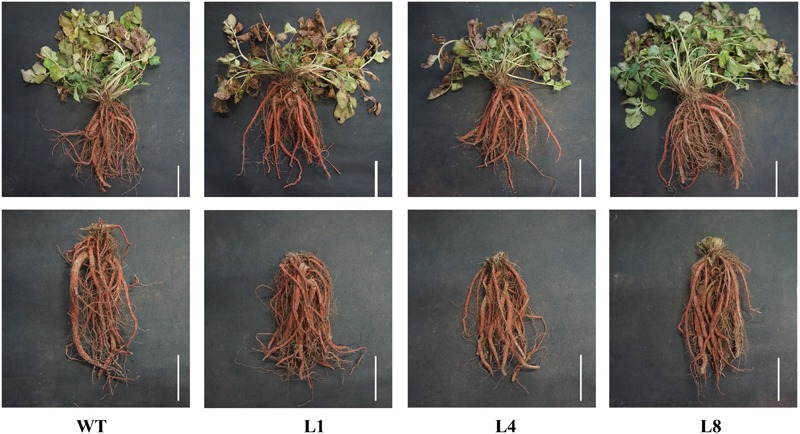
Appearance of representative whole plants (top) and roots (bottom) of WT and three transgenic lines of *S. miltiorrhiza*. The plantlets were transferred to the experimental field and grown for 5 months. Scale bar = 10 cm.

**FIGURE 9:**
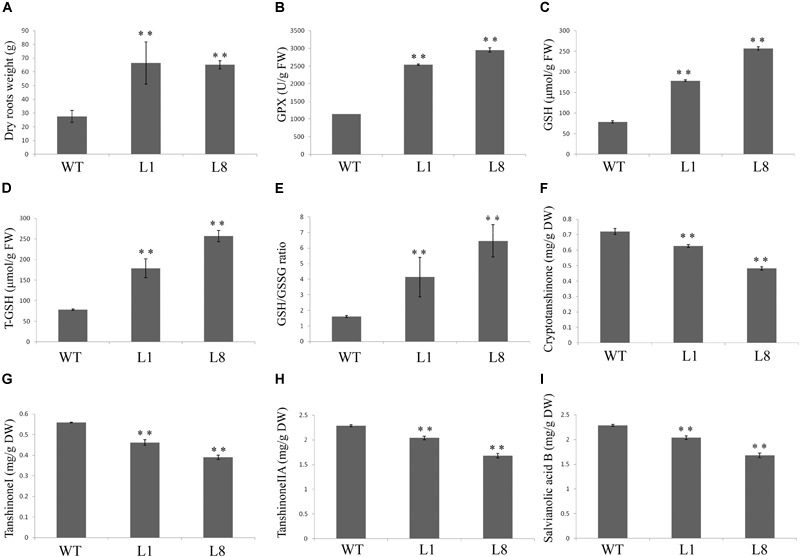
Physiological indices measured in roots of WT and two transgenic lines of *S. miltiorrhiza*. **(A)** Root weights after natural drying; **(B–E)** several indices of the glutathione cycle were measured in fresh roots. **(B)** Glutathione peroxidase (GPX) enzyme activity; **(C)** reduced glutathione (GSH) content; **(D)** total glutathione (T-GSH) content; **(E)** GSH/GSSG ratio. **(F–I)** The active ingredient contents were measured in dried roots. **(F)** cryptotanshinone; **(G)** Tanshinone I; **(H)** Tanshinone IIA; **(I)** Salvianolic acid B. Bars represent the mean ± SE of three independent experiments. ^∗∗^ represents significant differences at *p* < 0.01 compared with WT.

Compared with the controlled conditions in a growth chamber, natural environmental factors are complicated and changeable. To see whether the *RcGPX5* over-expressing lines showed better adaptability than WT, seedlings of similar size were planted in an experimental field and grown from June to November of 2017. There were no distinct differences in phenotype including the roots and above-ground parts between WT and the transgenic lines (Figure 8). However, the dry root weight was significantly higher (>2-fold) in the transgenic lines (Figure 9). In addition, we measured the GPX enzyme activity and GSH and total GSH contents of fresh roots to investigate the changes between the WT and transgenic lines caused by *RcGPX5* overexpression. We found that both GPX enzyme activity and GSH/T-GSH contents were also significantly higher in the transgenic lines than in WT (Figure 9).

### Overexpression of *RcGPX5* Decreases the Levels of Salvianolic Acids and Tashinones in the Roots of Transgenic *S. miltiorrhiza*

Although the root dry weights differed between the WT and transgenic plants, the appearance and color of the fresh rhizomes were similar. To investigate whether the production and accumulation of the active ingredients were changed by *RcGPX5* overexpression, we measured the contents of water-soluble salvianolic acids and lipid-soluble tanshinones in plants of WT and two transgenic lines (L1 and L8) according to the method given in the pharmacopeia (Figure 9). The results of this experiment showed that tanshinone content, which consisted of the three components tanshinone I (T-I), tanshinone IIA (T-IIA), and dihydrotanshinone I (DH-TI), was lower in the transgenic lines than in WT. We found the same situation for salvianolic acid (Sal B) content. In addition, the contents of all active ingredients were highest in WT and lowest in transgenic line 8, which was inconsistent with total GSH content and GPX enzyme activity (Figure 9). We interpret this to mean that the GSH content and/or GPX enzyme activity could have a negative relationship with respect to the accumulation of salvianolic acids and tashinones.

### Overexpression of *RcGPX5* Decreases Salvianolic Acid and Tashinone Content by Down-Regulating the Expression of Biosynthesis Genes

**FIGURE 10:**
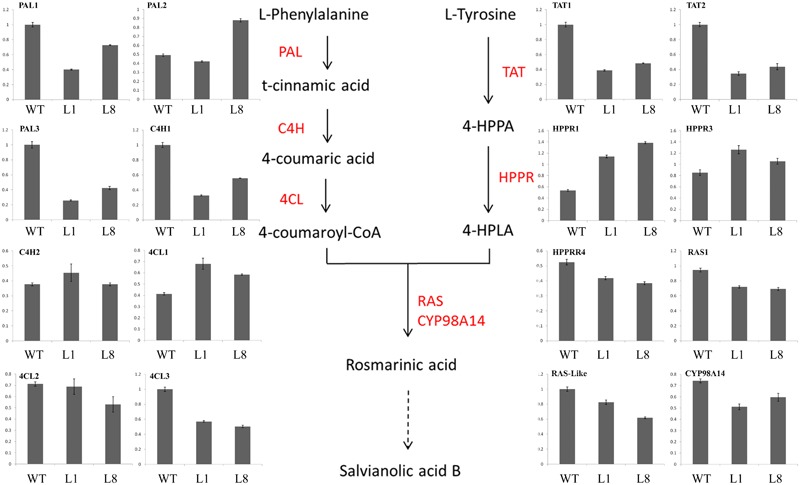
Expression of the salvianolic acid biosynthesis pathway genes in roots of WT and *RcGPX5* transgenic *S. miltiorrhiza* plants that were grown in the field for 5 months.

**FIGURE 11:**
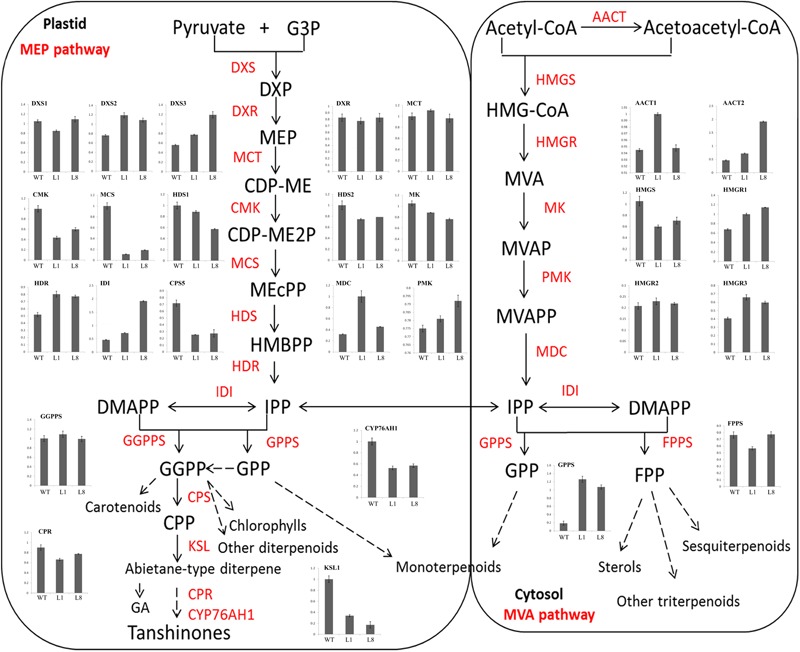
Expression of terpenoid biosynthesis pathway genes in roots of WT and *RcGPX5* transgenic *S. miltiorrhiza* plants.

Because we found decreased contents of salvianolic acids and tashinones in the transgenic plants, we next evaluated the relative expression levels of genes in two metabolic biosynthesis pathways by qRT-PCR. Total RNA (three biological replicates) was extracted from fresh roots of WT and transgenic lines L1 and L8. We examined the expression of 16 and 27 genes in the salvianolic acid and tashinone pathways, respectively (Figures 10, 11). Compared with WT, we found that upregulated and downregulated genes were present in the two pathways in both L1 and L8 plants. Of the 16 genes in the salvianolic acid biosynthesis pathway, 10 were distinctly downregulated in the transgenic lines (Figure 10). For the tashinone pathways, 10 of 27 genes were downregulated, 12 of 27 genes were upregulated, and 5 of 27 genes showed little or no changes in expression compared to WT. In particular, more genes showed downregulated expression in the plastid MEP pathway, while the opposite was found for genes in the cytosolic MVA pathway (Figure 11).

## Discussion

As a result of global warming, drought, heat, soil salinization, and desertification have increased in many parts of the world, and this trend is expected to continue. *S. miltiorrhiza* and other plants could therefore be exposed to increased levels of abiotic stress. Drought stress is one of the most adverse factors affecting plant growth and productivity, and can even cause significant declines in yield for *S. miltiorrhiza* (Anjum et al., 2011; Wei et al., 2016b). In order to adapt to water deficits, plants have developed elaborate mechanisms to survive drought stress and maintain growth and yield. These adaptive mechanisms play roles in many physiological and genetic processes such as plant structure, growth rate, photosynthesis, osmotic potential, and antioxidant defenses (Duan et al., 2007; Anjum et al., 2011). In addition, it possible to enhance drought tolerance in plants through genetic engineering. In *S. miltiorrhiza*, genetically modified plants can show distinctly better growth compared to WT through increased expression of endogenous or exogenous drought-associated genes (Han et al., 2007; Wu et al., 2014; Wei et al., 2016a; Wang et al., 2017). In this study, a GPX 5 (GPX5) gene from *R. crenulata* was introduced into the *S. miltiorrhiza* genome by Agrobacterium-mediated transformation. The resulting transgenic plants showed increased tolerance to H_2_O_2_ and drought treatment due to the consistently high levels of expression of *RcGPX5*, and the materials could be helpful to molecular function validation of *RcGPX5* and to improve the agronomic traits of *S. miltiorrhiza*.

Reactive oxygen species are produced naturally during photosynthesis and respiration through the electron transport chains. Furthermore, abiotic and biotic stresses can also dramatically increase ROS production and accumulation. Other than their roles in oxidative stress, ROS function as signaling molecules or alter the oxidative cellular environment to influence the expression of downstream genes and regulate organismic development (Munne-Bosch et al., 2013). Thus, the generation and scavenging of ROS could be a deliberate progress. In plant cells, the lifetime of ROS is mainly determined by catalases and enzymes of the glutathione-ascorbate cycle (Gueta-Dahan et al., 1997). Within the antioxidant network, GPX can reduce H_2_O_2_ and phospholipid hydroperoxides at the expense of GSH by using thioredoxin (Trx) as the electron donor (Gelhaye et al., 2004). In this study, leaves of transgenic *S. miltiorrhiza* plants expressing *RcGPX5* had stronger tolerance to H_2_O_2_ stress than WT. Exogenous H_2_O_2_ destroys the cell biomembrane lipid layers, which induces the accumulation of MDA (from lipid peroxidation) and causes intracellular water loss. In addition, H_2_O_2_ also disrupts the chloroplast membrane and its endomembrane systems, causing leakage of photosynthetic pigments (Figure 3). Due to the high level of *RcGPX5* expression, leaves of transgenic lines are able to scavenge more H_2_O_2_ and maintain the cell membrane structure. In addition, overexpression of *RcGPX5* also confers drought tolerance to the transgenic plants. In the case of persistent water deficits, WT plants wilted earlier and died at the later stages of drought stress treatment. However, the *RcGPX5* transgenic lines were able to recover and grow normally soon after re-watering 13 days after water was initially withheld. During this process, we found that the content of glutathione, including total glutathione, reduced glutathione, and oxidized glutathione, changed markedly in the transgenic plants. Compared with WT prior to drought treatment, the T-GSH contents and the GSH/GSSG ratios in the transgenic plants increased, and the GSH contents were less than in WT, which had a high GSH/GSSG ratio. After exposure to drought, even though the T-GSH content increased sharply in WT, the GSH content was higher in the transgenic lines, which also had higher GSH/GSSG ratios. This could be explained by an increase in the rate of glutathione generation and oxidation due to *RcGPX5* over-expression. This inference can be clarified by examining the activities of the major enzymes of the glutathione-ascorbate cycle (GPX, GR, and APX) under control and drought conditions (Figure 5). Moreover, the expression levels of key genes involved in glutathione synthesis and the glutathione-ascorbate cycle also provide evidence to support this; for example, *GSH II, APX, GR*, and *MDHAR* all show higher relative expression in the transgenic lines under control conditions (Figure 6). All the changes caused by *RcGPX5* expression in the transgenic lines could define their potential ability to tolerate drought (Roxas et al., 2000). In addition, although the mechanism of plant GPX molecular function is complicated and is not well characterized, it is well known that GPX can interact directly or indirectly with other proteins and represents a putative link between the glutathione-based and the thioredoxin systems (Szalai et al., 2009; Bela et al., 2015). Thus, the improved drought tolerance observed in the *RcGPX5*-overexpressing lines could be partly attributed to an increase in the level of *SmABI2* gene expression by physical interaction and its participation in the ABA signaling pathway, which has been shown to directly interact with *AtABI2* in the yeast two-hybrid assay (Zhang et al., 2018). Also, more remarkably, expression of a transcription factor gene, *SmCBF*, which is related to multiple abiotic stresses, was also induced in the transgenic lines. However, the link between *RcGPX5* and *SmCBF* will require further study. In addition, the reason behind the changes in expression of other groups of genes resulting from *RcGPX5* over-expression is presently unknown, although these genes could conceivably participate in multiple pathways to protect the plant from drought damage.

Plant GPX enzymes contain three Cys active sites and catalyze the reduction of H_2_O_2_ and other organic peroxides by consuming two glutathiones. In this reaction, the reduced glutathiones are transformed to oxidized GSSG (Koh et al., 2007). In the transgenic lines, high expression levels of *RcGPX5* could increase the rate of the transformation reaction, resulting in a low level of GSH. And the reduced GSH feedback regulates the glutathione synthesis pathway, which enlarges the size of the T-GSH pool. One result of this study is that the T-GSH pool under control conditions was much larger in the transgenic lines than in WT. On the contrary, the H_2_O_2_ and O_2_^-^ contents, along with the SOD enzyme activity, were all higher in the transgenic lines under control condition (Figure 5). It might be a very bold assumption that even though in a favorable environment, more H_2_O_2_ was needed than in *RcGPX5* over-expression transgenic plantlets than in the wild type, which could trigger the organism to produce ROS by electron transport chain or some other metabolism mechanism. However, despite this assumption, the transgenic seedlings did not have different phenotypes compared to WT under normal conditions, even though they had lower GSH/GSSG ratios and higher expression levels of genes associated with photosynthesis (Figure 1A).

Recently, with more studies being published on the potential pharmacological activities of *S. miltiorrhiza*, the demand has increased steadily, and it has been estimated that China needs 20 million kilograms per year (Liu et al., 2011). Therefore, it is necessary to increase the yield of *S. miltiorrhiza* roots (Figure 9A). In this study, we found that plants over-expressing *RcGPX5* produced more harvestable dry roots after 5 months of growth in the field. However, the levels of two particular active secondary metabolic products, the salvianolic acids and tanshinones, were decreased in the transgenic roots (Figures 9F–I). In addition some gene expression levels were decreased, such as for PAL, TAT, C4H1, 4CL2/3, HPPRR4, RAS1, and CYP98A14 in the salvianolic acid biosynthesis pathway and CMK, MCS, HDS, MK CYP76AH1, and HMGS in tanshinone biosynthesis pathways. These genes with lower expression levels finally reduced the accumulation of secondary active ingredients in transgenic lines (Figures 10, 11). And there have been reports demonstrating that these secondary metabolites could potentially protect the cells from oxidative injury (Matkowski et al., 2008; Zhao et al., 2008). Accumulation of secondary metabolites is known to be a defense mechanism for plants as they face various environmental challenges (Liu et al., 2011). The region where *S. miltiorrhiza* is grown is in the north of China, which has a temperate monsoon climate with lower temperatures but suffered from drought in late November of 2017. As a result of *RcGPX5* over-expression, transgenic plants of *S. miltiorrhiza* may have increased tolerance to multiple abiotic or biotic stresses, and they have a less oxidizing cellular environment, which decreases the expression levels of genes in the salvianolic acid and tanshinone biosynthesis pathways. However, whether a competing relationship exists for the glutathione pathway and secondary metabolite biosynthesis pathways will require additional study in the future.

In this paper, the GPX *RcGPX5* was introduced into *S. miltiorrhiza* and obtained high expression level transgenic lines. Furthermore, drought and H_2_O_2_ tolerances for wild-type and transgenic lines were analyzed. After 5 months, we also found transgenic lines held higher dry weight and GSH contents. The result indicated that *RcGPX5* should be a competitor with secondary metabolic for *S. miltiorrhiza* to respond to abiotic or biotic stress. While the processes of commercializing transgenic *S. miltiorrhiza* in the future might require a lot of costs, *RcGPX5* provides an alternative gene to improve tolerance of drought or other abiotic stresses for *S. miltiorrhiza*. In future research, the selective marker gene (Basta) could be cleared away by Crispr-Cas9 technology or by crossing and back-crossing with the wild type. These strategies might accelerate the process of transgenic *S. miltiorrhiza* commercialize.

## Conclusion

In the research presented here, we analyzed the function of the *R. crenulata glutathione peroxidase 5* (*RcGPX5*) gene by ectopic expression in *S. miltiorrhiza*. The results showed that over-expression of *RcGPX5* can confer tolerance to H_2_O_2_ and drought in *S. miltiorrhiza*. The transgenic lines showed higher levels of reduced glutathione, total glutathione, and increased activities and gene expression levels for several enzymes in the glutathione-ascorbate cycle. *RcGPX5* may participate in multiple pathways such as abscisic acid signaling through transcription factors by increasing the gene expression levels under control conditions, which in turn provides tolerance to oxidative stress. In addition, the dried roots from the transgenic lines had increased biomass after 5 months growing in the field, which demonstrates that *RcGPX5* could play an important role in the plant response to many environmental stimuli. However, the levels of several secondary metabolites in the roots were lower than in WT, and the relative expression of genes involved in the biosynthesis of salvianolic acids and tashinones was reduced in the transgenic lines. Thus, *RcGPX5* might act as a competitor with the secondary metabolites in response to environmental stimuli in *S. miltiorrhiza*.

## Author Contributions

LZ, MW, YT, CC, and SJ designed the experiments, analyzed the data, and wrote the manuscript. LZ and MW performed the main experiments. YT and SJ contributed to the abiotic stress experiments. DY and TW made a significant contribution to the manuscript. WS responsible for the manuscript design and validation analysis.

## Conflict of Interest Statement

The authors declare that the research was conducted in the absence of any commercial or financial relationships that could be construed as a potential conflict of interest.

## Supplementary Material

FIGURE S1 *SmACTIN* and *RcGPX5* gene expresslion levels in WT and transgenic lines. **(A)** qRT-PCR analysis of T0 transformants using quantified primers for RcGPX5 in WT and transgenic lines 1–8. **(B)** *RcGPX5* tissue-specific expression in *S. miltiorrhiza*.

TABLE S1 Annotation information for selected unigenes identified in the *S. miltiorrhiza* transcriptome.

TABLE S2 Nucleotide sequences of primers used for real-time PCR of genes identified in the *S. miltiorrhiza* transcriptome.
