# Supplementary figures and images for: Overexpression of the Glutathione Peroxidase 5 (RcGPX5) Gene From Rhodiola crenulata Increases Drought Tolerance in Salvia miltiorrhiza
Source: Front Plant Sci. 2019 Jan 9;9:1950. doi: 10.3389/fpls.2018.01950 (PMC6333746; doi:10.3389/fpls.2018.01950)

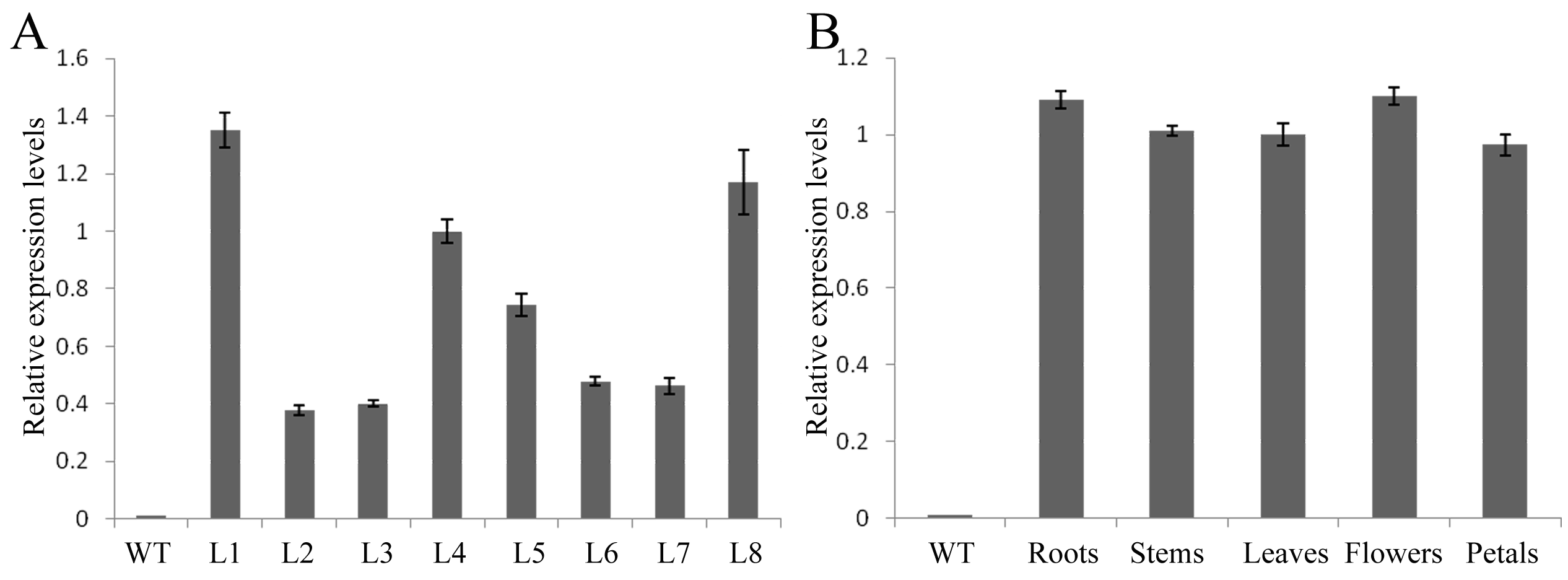

Supplement: FIGURE S1 — SmACTIN and RcGPX5 gene expresslion levels in WT and transgenic lines. (A) qRT-PCR analysis of T0 transformants using quantified primers for RcGPX5 in WT and transgenic lines 1–8. (B) RcGPX5 tissue-specific expression in S. miltiorrhiza. [file Image_1.TIF]
